# Illness Perception, Emotional Distress, and Obsessive–Compulsive Symptomatology in Patients with Alopecia Areata: A Mediation Study

**DOI:** 10.3390/bs16010092

**Published:** 2026-01-09

**Authors:** Tonia Samela, Francesco Moro, Giorgia Cordella, Valeria Antinone, Maria Beatrice Pupa, Jo Linda Sinagra, Damiano Abeni, Laura Colonna

**Affiliations:** 1 Clinical Psychology Unit, Istituto Dermopatico dell’Immacolata, IDI-IRCCS, 00167 Rome, Italy; g.cordella@idi.it (G.C.); v.antinone@idi.it (V.A.); 2 Clinical Epidemiology Unit, Istituto Dermopatico dell’Immacolata, IDI-IRCCS, 00167 Rome, Italy; m.pupa@idi.it (M.B.P.); d.abeni@idi.it (D.A.); 3 Clinical Dermatology Unit, Istituto Dermopatico dell’Immacolata, IDI-IRCCS, 00167 Rome, Italy; f.moro@idi.it (F.M.); j.sinagra@idi.it (J.L.S.); l.colonna@idi.it (L.C.)

**Keywords:** Alopecia Areata, psychodermatology, patient reported outcome measures, illness perception, obsessive–compulsive symptomatology

## Abstract

Alopecia Areata (AA) is a chronic disorder with significant psychological impact due to its unpredictability. While emotional distress (ED) is well-recognized in AA, the interplay between illness perception (IP) and obsessive–compulsive (OCD) symptomatology remains underexplored. This -sectional, observational study aimed to investigate the prevalence of OCD symptoms and ED in AA outpatients, analyzed the relationship between IP and OCD symptomatology. One-hundred-thirty-five AA outpatients, from a specialized Hospital in Rome, Italy, were recruited. Participants completed the DASS-21 for ED, the Brief IPQ for IP, and the OCI-R for OCD symptomatology. AA severity was assessed using standardized scores. Statistical analyses included correlations and a simple mediation model. OCD symptomatology was found in 18.5% of the sample, and clinical-level ED in 20.7%. Strong associations were found between OCI-R and DASS-21 (r = 0.56, *p* < 0.001), and DASS-21 and Brief IPQ (r = 0.56, *p* < 0.001). The mediation analysis indicated that ED fully mediated the relationship between IP and OCD symptomatology (indirect effect: b = 0.20, 95% CI [0.10, 0.30]), suggesting IP’s impact on OCD symptoms primarily occurs via ED. Negative IP exacerbate ED, which, in turn, drives OCD behaviors. Psychological assessment and targeted interventions in individuals with AA are needed.

## 1. Introduction

Alopecia Areata (AA), a chronic autoimmune disorder characterized by non-scarring hair loss, affects approximately 20.2 per 100,000 person-years, without significant sex differences (Villasante Fricke & Miteva, 2015). Lifetime risk estimates are 1.7–2%, mainly before age 30 (H. H. Lee et al., 2020). AA pathogenesis involves genetic predisposition and external triggers (Bertolini et al., 2020; Petukhova et al., 2010). AA presents in diverse forms, from localized patches to alopecia universalis (Pelzer & Iorizzo, 2024; Roest et al., 2018; Zhou et al., 2021). Diagnosis is primarily clinical, supported by dermoscopy and, if necessary, histopathology (Olsen et al., 2004).

Moreover, AA is associated with several comorbidities (e.g., atopic diseases, metabolic syndromes, thyroid diseases) (S. Lee et al., 2019) and recurrence rates are high (Lyakhovitsky et al., 2019; You & Kim, 2017). Treatment depends on clinical severity and patient age. For limited disease, first-line therapeutic options include topical or intralesional corticosteroids, whilst, for extensive involvement, systemic therapies such as systemic corticosteroids, immunotherapy, or methotrexate are preferred. Emerging treatments, particularly JAK inhibitors, are now widely used and first-line treatment in chronic AA with Severity of Alopecia Tool (SALT) scores > 50% (Lintzeri et al., 2022; Rudnicka et al., 2024).

The management of chronic skin and hair disorders often necessitates an integrated approach, highlighting the importance of the field of Psychodermatology. This interdisciplinary specialty concerned with the interaction between psychological functioning and skin, addressing conditions where psychological distress is either the cause, the consequence, or an exacerbating factor of dermatological diseases (Ferreira et al., 2024; Picardi et al., 2005). According to the recent proposal for an international classification of psychodermatological disorders, AA is categorized under the group of conditions where Psychological Factors Impact on Skin Diseases (PFSID), underscoring the strong reciprocal relationship between psychological status and disease severity. Notably, AA must be differentiated from Trichotillomania, which is classified as an Obsessive–Compulsive Related Disorder (OCRD) according to the Diagnostic and Statistical Manual of Mental Disorders (DSM-5) and the International Classification of Diseases (ICD-11). Differently from AA, TTM involves repetitive hair pulling resulting in hair loss, driven by emotional or cognitive factors, and is categorized within psychodermatology spectrum as a Primary Psychiatric Disorder with Dermatological Manifestations (PPDDM) (Ferreira et al., 2024).

The chronic, unpredictable and visible nature of AA provides fertile grounds for anxiety (Caldarola et al., 2024; Macbeth et al., 2022; Rencz et al., 2016), as the uncertainty related to disease flares could engenders chronic stress where patients may experience feelings of helplessness and an inability to control their condition (Mesinkovska et al., 2023). This chronic burden, in turn, may trigger emotional distress (ED) (Burns et al., 2020; Caldarola et al., 2024). Notably, the visible nature of AA could lead patients to be particularly susceptible to perceived stigmatization. The perceived social isolation, judgment, and devaluation associated with this visible chronic condition, contributes substantially to ED and reduced Quality of Life (QoL) (Van Beugen et al., 2023).

Moreover, a central role in fostering ED is also played by illness perception (IP), which refers to the organized cognitive representations or beliefs that patients have about their illness. Different studies have highlighted how negative IP, such as a strong illness identity and perceived severe consequences, correlate with poorer QoL, anxiety, depression, and heightened ED (Jaltuszewska et al., 2023; O’Connor et al., 2023). In fact, IP have been found to be an important determinant of behavior and have been associated with a number of important outcomes, such as treatment adherence, functional recovery, and QoL (Foxwell et al., 2013; Leventhal et al., 1997; Solmaz et al., 2021). In fact, a study conducted by Firooz et al. (2005) revealed that a substantial proportion of patients believed their illness had significant repercussions on their lives and that it was a permanent rather than a transient condition, with just over half of the patients considered their treatments to be effective. Additionally, Nasimi et al. (2021) highlighted significant negative effects of the illness on patients’ emotions, in fact, over 80% of the participants of that study reported feeling upset when thinking about their condition, and more than 70% experienced symptoms of ED (Nasimi et al., 2021). Furthermore, 66% of patients acknowledged the consequences of the illness as relevant in their lives, perceiving a considerably negative impact.

A negative IP and an high ED likely interact in a reciprocal manner, producing a self-perpetuating cycle (Mostaghimi et al., 2021). This distress encompasses a range of emotions including sadness, anxiety, anger, and frustration, feelings of embarrassment, stigma, or diminished self-esteem, creating an ideal substrate for the development of obsessive thoughts and compulsive behaviors (Sookman & Pinard, 2002; Toussi et al., 2021).

Obsessive–Compulsive Disorder (OCD) is a psychiatric condition, with a lifetime prevalence estimated in adult population about 1.3% Fawcett et al. (2020) and it is characterized by uncontrollable obsessive thoughts and compulsive behaviors. Particularly, obsessions are persistent and recurrent preoccupations, images, impulses or urges are experienced as unwanted, distressing, and intrusive (Cervin, 2023), while compulsions consist of repetitive behaviours and/or mental acts that the individual feels driven to perform in response to an obsession, according to rigid rules, or to achieve a sense of ‘completeness’ (Bokor & Anderson, 2014; Harrison et al., 2021).

In this study, our primary objective is to hypothesize that IP could serve as the initial predictor of OCD symptomatology. Specifically, when AA patients perceive their illness as having a severe timeline and high personal consequences (i.e., poor IP), this cognitive representation directly exacerbates their ED. This ED—characterized by chronic anxiety and worry—then acts as the mediator, guiding the initial negative IP into the more structured and debilitating pattern of OCD symptomatology.

In other words, the distinction between AA general mental health burden and its specific link to OCD symptomatology is rooted in the type of distress the illness imposes. While the general mental health impact encompasses a broad array of negative emotions like sadness, frustration, and anxiety, resulting directly from the visible and stigmatizing nature of the condition (ED), the vulnerability to OCD spectrum symptoms arises more specifically from the unpredictable and chronic uncertainty of AA. This unpredictability—the inability to forecast when or where hair loss will occur, or if treatment will be effective—fosters a state of chronic anxiety. This chronic anxiety, coupled with an underlying intolerance of uncertainty, creates the psychological precondition for obsessive–compulsive features. Such features are inherently driven by a need to reduce anxiety and regain control through ritualistic thought or behavior. Therefore, AA does not just cause general mood issues, but its unique characteristic as an uncontrollable, relapsing disorder specifically heightens the vulnerability to developing or exacerbating this spectrum of anxiety-driven control mechanisms. For instance, rigid thought patterns (typical of OCD) can interfere with the flexibility required for adapting to long-term medical treatments, and high levels of anxiety can exacerbate the hair-loss cycle, lowing adherence to complex dermatological and systemic treatment protocols.

While psychiatric disorders that mimic “(e.g., hair-pulling disorder; Alopecia Factitia; Trichoteiromania) have been relatively well-explored in the literature (Mavrogiorgou et al., 2015), few studies have investigated the coexistence of OCD symptomatology in AA. Among the limited studies available on the topic, Chu et al. (2012) conducted a nationwide case–control study in Taiwan and found that patients with AA aged 40 to 59 years showed the highest odds of OCD compared to controls (OR 3.00; 95% CI 1.11–8.12). Moreover, a study by Talaei et al. (2017) found that AA patients exhibited higher OCD symptomatology compared to controls. Furthermore, patients with a history of AA relapse reported higher OCD symptomatology than those without relapse, suggesting that AA patients with recurrences are more likely to experience OCD symptoms.

### Aims

The available literature has yet not comprehensively investigated the complex interplay between IP, ED, and the manifestation of OCD symptoms in individuals affected by AA. Understanding these psychological mechanisms is essential for developing targeted clinical interventions. Therefore, the present cross-sectional, observational study aims at contributing to understand this interplay, fulfilling two primary objectives: (i) to investigate the presence of OCD symptomatology and ED among outpatients diagnosed with AA, and (ii) to analyze how Negative IP, in conjunction with ED, potentially exacerbates OCD symptomatology, thereby worsening the mental health and QoL of people affected by AA.

## 2. Materials and Methods

### 2.1. Participants

This cross-sectional, observational study was approved by the Istituto Dermopatico dell’Immacolata IDI-IRCCS Ethical Committee (Prot.N. 690/1). The research was conducted in compliance with the Helsinki Declaration. Data were collected from October 2023 to April 2024, in the setting of the Second-level outpatient clinic dedicated to AA of the IDI-IRCCS, Rome, Italy. Participants were consecutively enrolled at the time of their initial clinical visit. Only patients who agreed to participate and provided written informed consent were included in the study. Inclusion criteria were: (i) 18+ years of age; (ii) AA diagnosis confirmed by dermatological examination; (iii) ability to complete questionnaires. Exclusion criteria were: (i) past or current diagnosed psychiatric disorders; prior or concomitant use of psychotropic medication; (ii) inability to complete assessment (i.e., linguistic barriers, illiteracy, neuropsychological conditions). Participants received no honorarium or any benefit from participating in the study. No participants withdrew from the study during the questionnaire completion process.

### 2.2. Measures

*AA severity*. A standardized form was completed by clinicians regarding disease severity (i.e., SALT; Physician Global Assessment -PGA-). The SALT total score categorizes AA severity into five groups from 0% (no hair loss), to 100% (total scalp hair loss) (Olsen et al., 2004). The PGA is a simpler, clinician-rated scale used to provide an overall severity grade based on clinical impression.

Participants also completed a standardized form with sociodemographic information [e.g., age; sex; weight and height in order to calculate the Body Mass Index (BMI); marital status; education level)], and were administered the Italian versions of Depression Anxiety Stress Scale (DASS-21); the Brief Illness Perception Questionnaire (Brief IPQ); the Obsessive–Compulsive Inventory Revised Form (OCI-R).

*General psychopathology*. The DASS-21 is a self-report measure of depression, anxiety, and stress. It has 21 items on a 4-point Likert scale, with higher scores indicating greater distress (Bottesi et al., 2015). Cronbach’s α in this sample was 0.94. In this study, ED was operationalized as the Total Score obtained from the DASS-21. Following established psychometric practice in clinical and psychological research, the DASS-21 Total Score (the summation of all 21 items) was utilized as a global, single-factor index of negative affect, thereby representing the overarching construct of general ED (Caravaca-Sánchez et al., 2025; Henry & Crawford, 2005; Osman et al., 2012; Samela et al., 2021).

*Illness perception*. The Brief IPQ assesses how individuals perceive and manage illness consequences. It has 9 items (0–10 Likert scale), with higher scores indicating poorer adaptation (Pain et al., 2006). Cronbach’s α was 0.61. The overall instrument yielded a marginal coefficient (α = 0.61). While this value falls below the commonly accepted threshold for robust reliability, it is consistent with previous findings in diverse patient populations using the Brief IPQ (Emilsson et al., 2020; Hallegraeff et al., 2013). Furthermore, the Brief IPQ is a multi-dimensional tool intended to measure distinct, albeit related, cognitive and emotional components of illness perception; a lower overall α is observed and considered acceptable for brief, heterogeneous scales (Streiner, 2003).

*Obsessive/Compulsive symptoms*. The OCI-R, an 18-item (0–4 Likert scale) tool, measures distress from obsessions and compulsions. Higher scores indicate significant impairment. For the OCI-R, a cut-off of 21 (corresponding to the 91st percentile) was utilized to categorize participants as having a probable clinical level of symptomatology, following the established validation criteria by Marchetti et al. (2010). Cronbach’s α for this scale was 0.86.

### 2.3. Potential Sources of Bias

The observational and cross-sectional nature of this study, while appropriate for exploring the hypothesized relationships, necessitates the acknowledgement of potential sources of bias. Participants were recruited via convenience sampling from a specialist tertiary care center of the IDI-IRCCS of Rome. Patients seeking care at such a highly specialized clinic may present with more severe conditions (e.g., clinical extension or psychological impact) compared to the general population of AA patients. This approach limits the external validity of the findings. Furthermore, the exclusion of individuals with a history of psychiatric disorders may have resulted in a sample with potentially lower levels of psychopathology. Moreover, our reliance on self-report measures for psychological variables (i.e., IP, ED, and OCD Symptomatology) is a source of information bias. This approach carries the risk of social desirability bias and recall bias, which could affect the accuracy of the reported symptoms. We attempted to mitigate this by ensuring anonymity and confidentiality.

### 2.4. Statistical Analysis

All the analyses were performed with the Statistical Package for the Social Sciences (SPSS) 28.0 (IBM Corp, 2019). All statistics were considered significant for *p* < 0.05. Data were described as numbers, percentages, and frequency rates. Differences in the distribution of categorical variables were assessed by comparing the absolute frequencies using the Chi-Square test for independence. Where necessary, Fisher’s Exact Test was employed. To examine the preliminary associations between the study’s continuous variables, Pearson’s correlation coefficient was utilized (Baron & Kenny, 1986; Cohen, 1977; Hemphill, 2003). A simple mediation model with a single mediator (model no.4) was tested, using the PROCESS V3.5 macro for SPSS (Hayes, 2013). Unstandardized coefficients (b) and 95% bootstrap confidence intervals (CI) (5000 samples) were reported (Hayes, 2013). The key effects estimated within the simple mediation model are defined as follows: the Total Effect (c) represents the overall, unadjusted relationship between the predictor and the outcome. It is the sum of the direct and indirect effects (c = c’ + b) and is calculated in a regression model where the predictor is the sole independent variable predicting the outcome; while the Direct Effect (c’) represents the relationship between the predictor and the outcome after controlling for the mediator; The Indirect Effect (paths “b”) quantifies the influence of the predictor on the outcome through the mediator; it is computed as the product of paths.

According to Preacher and Hayes (2008), confidence intervals that do not include zero could provide evidence of a significant indirect effect. Even though mediation models cannot identify causal relationships between variables when data from correlational studies are used (Maxwell et al., 2011) these techniques can be effective in analyzing whether a third variable could mediate the relationship between two other variables.

## 3. Results

One-hundred thirty-five patients (96 women) participated. Twenty-five participants (18.5%) scored above the cut-off of 21 on the OCI-R, indicating a probable clinical level of OCD symptomatology in this subgroup.

The analysis of total score of the DASS-21 in our sample reveals a considerable psychological burden. The mean DASS-21 Total Score (M = 18.66) is significantly higher than the average reported for the non-clinical normative population (M = 12.3) (Bottesi et al., 2015). This score places the average patient in our sample within the moderate range of ED, confirming the clinical relevance of this variable. While our mean score is slightly lower than the average found in the clinical validation sample (M = 22.1) (Bottesi et al., 2015), the high standard deviation (SD = 26.65) suggests considerable heterogeneity within the AA population, indicating the presence of a substantial subgroup experiencing severe-to-extremely severe distress. This finding aligns with the fact that 18.5% of our participants showed probable clinical levels of OCD symptomatology. Moreover, 98.5% of the sample declared to have onset or maintenance insomnia. Finally, the mean SALT level measured in this sample was 50.12 (±36.41). For an extensive description of socio-demographic and clinical features of the sample see Table 1.

No statistically significant associations were found among sociodemographic features (i.e., age, BMI) and clinical severity. No gender differences were found in mean scores among principal outcomes (IPQ: t_74.91_ = −0.017, *p* = 0.63; DASS-21: t_73.42_ = 0.097, *p* = 0.84; OCI-R: t_64.29_ = 0.046, *p* = 0.20), and among clinical severity indexes (i.e., SALT: t_−1.25_, *p* = 0.975; PGA: t_−1.29_, *p* = 0.101). A significant, small positive relationship was observed between age and BMI (r = 0.280, *p* < 0.01). Except for this finding, the sociodemographic variables (did not demonstrate significant correlations with any of the clinical variables (SALT, DASS-21 scales, B-IPQ, and OCI-R). The SALT measure also failed to show any significant correlation with any other variable included in the analysis. The analysis revealed several strong, expected intercorrelations among the DASS-21 subscales, on fact the total score was significantly and positively correlated with Anxiety subscale (r = 0.541, *p* < 0.01), Depression subscale (r = 0.658, *p* < 0.01), and Stress subscale (r = 0.301, *p* < 0.01). DASS-21 Anxiety subscale and DASS-21 Depression subscale were also strongly associated (r = 0.411, *p* < 0.01). DASS-21 Stress subscale was only significantly correlated with the DASS-21 total score and showed non-significant associations with Anxiety (r = 0.054) and Depression subscale (r = 0.088). The B-IPQ-R demonstrated significant positive correlations with DASS-21 total score (r = 0.354, *p* < 0.01) and DASS-21 Depression subscale (r = 0.180, *p* < 0.05). Finally, the OCI-R score was positively and significantly correlated with the DASS-21 total score (r = 0.559, *p* < 0.01), DASS-21 Anxiety subscale (r = 0.233, *p* < 0.01), DASS-21 Depression subscale (r = 0.383, *p* < 0.01), and B-IPQ-R (r = 0.251, *p* < 0.01) (Table 2).

**Figure 1:**
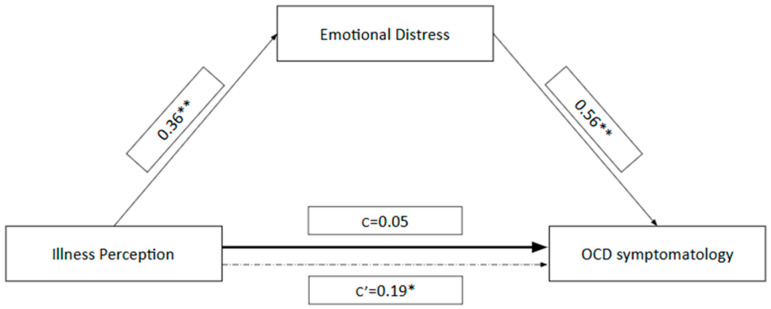
Mediation Analysis of Illness Perception, Emotional Distress, and OCD Symptomatology. ** *p* < 0.01; * *p* < 0.05.

In the mediation model, Brief-IPQ was the independent variable, OCI-R the dependent, and DASS total score was set as the mediator (Figure 1). The model accounted for 13% of the variance in score (*F*_1;128_ = 18.93, *p* < 0.001). The total effect (*c* = 0.22, SE = 0.08, *t* = 2.93, *p* < 0.001) of B-IPQ score on OCI-R score was significant, whilst the direct (*c*′ = 0.05, SE = 0.07, *t* = 0.75, *p* = 0.450) was not, indicating that the mediator has more impact on the OCD symptomatology compared to IP. The indirect effect of B-IPQ score on OCI-R score through DASS total score was also significant (*b* = 0.19, SE = 0.05, 95% bootstrap CI [0.10, 0.30]), indicating that B-IPQ score influenced OCI-R score indirectly via DASS-21 score.

Mediation model depicting the hypothesized relationship between the Brief Illness Perception Questionnaire-Revised (B-IPQ-R) score (Independent Variable, IV), the Obsessive–Compulsive Inventory-Revised (OCI-R) score (Dependent Variable, DV), and the Depression Anxiety Stress Scales-21 (DASS-21) score as the Mediator. Path values represent standardized regression coefficients (β). The direct effect of Illness Perception on OCD Symptomatology is denoted by Path c’ (after inclusion of the mediator). Path c represents the total effect of Illness Perception on OCD Symptomatology (before mediator inclusion). The indirect effect, mediated by Emotional Distress, is reported with a 95% Confidence Interval (CI) obtained from 5000 bootstrap analyses. * *p* < 0.05 significant effect; ** *p* < 0.001 significant effect.

## 4. Discussion

This study investigated IP and OCD symptomatology in AA, focusing on the mediating role of ED. While the prevalence of OCD symptoms has been explored in various dermatological patient populations, their specific occurrence in AA outpatients remains underexplored. For example, Demet et al. (2005) conducted a study on 166 dermatological patients revealing that 24.7% met the DSM-IV criteria for OCD, yet AA was notably absent from their diverse sample, which included conditions such as acne, psoriasis, and various infections. Similarly, previous studies examining OCD prevalence in dermatological samples by Sheikhmoonesi et al. (2014), Fineberg et al. (2003), and Afkham Ebrahimi et al. (2007) did not specifically focus on patients with AA. While a systematic review and meta-analysis by Okhovat et al. (2023) confirmed an elevated risk of anxiety and depression among patients with AA, findings corroborated by the meta-analysis of Lauron et al. (2023), who reported a significantly higher prevalence rate for depressive and anxiety disorders compared to the general population (9% vs. 3.8% and 13% vs. 7.3%, respectively), the literature regarding OCD symptomatology remains scarce and inconsistent. Our current findings partially align with these observations, as we identified clinically relevant depressive and anxiety symptomatology in 6.8% and 14.9% of our sample, respectively.

However, a crucial finding is that 18.5% of our sample exceeded the OCI-R cut-off for OCD symptomatology, a result that notably diverges from the findings of Lauron et al. (2023), who did not detect OCD symptomatology in AA patients. In sum, there is a paucity of research and discordance among results concerning the prevalence of OCD symptomatology specifically in individuals with AA, despite the literature highlights the presence of such symptomatology across other dermatological settings. This research gap may stem from the relative infrequency of extensive psychological assessments for AA patients during the routine dermatological clinical practice, a finding that strongly corroborates the existing recommendation advocating for the integration of clinical psychology interventions in hospital dermatology practice (Aguilar-Duran et al., 2014; Finlay et al., 2021; Panebianco et al., 2018; Samela et al., 2022).

Concerning subgroup analysis, no statistically significant differences were found in IP, ED, OCD symptomatology among genders. These results are in contrast with those by Mesinkovska et al. (2023) and Titeca et al. (2020) who found that women with AA had consistently lower scores in QoL, and significantly higher scores in anxiety and depression scales than males.

Our data highlight a significant total effect of the Brief-IPQ (B-IPQ) on the OCI-R score, and a significant indirect effect through the DASS total score. However, the direct effect of B-IPQ on OCI-R after accounting for the DASS score is not significant. This pattern of findings strongly indicates full mediation among these variables. In other words, the relationship between IP and OCD symptoms is primarily explained through the mediating effect of ED.

A critical step in interpreting our findings involved a comparative analysis with the established literature, specifically referencing the seminal IPQ-R data reported by Cartwright et al. (2009). Given the difference in measurement metrics (B-IPQ: 0–10 scale; IPQ-R: 1–5 scale), we utilized a Linear Rescaling (Affine Transformation) (Y = (X − 1) × 2.5) (Kline, 1993) on the published IPQ-R means to ensure metrical comparability across domains. This standardization procedure maps the 1-to-5 scale onto the 0-to-10 scale proportionally, maintaining the relative position of the means across the two instruments, and thus allowing for a valid cross-study comparison of the IP domains (Kline, 1993; Weinman et al., 1996). This standardization procedure revealed several congruencies and distinctions, strengthening the conclusion that the high impact and low control are core, enduring features of the illness experience in AA patients. In the high-impact domains, the rescaled “Consequences” score from the earlier study converts to 6.4, which is only marginally lower than our reported mean of M = 7.1 (SD = 2.4). Similarly, the rescaled mean for “Illness Concern” converts to 5.17, yet this remains notably lower than our observed mean of M = 7.2 (SD = 2.6). These comparisons indicate a consistently high perception of severe consequences across both samples, but critically suggest that our sample perceived the disease as generating significantly greater current worry and concern. This heightened concern may reflect changing in clinical practices or increased awareness regarding long-term morbidity. Conversely, the converted mean for “Emotional Representation” in Cartwright et al.’s study is 6.9 (from 3.76 ± 1.0), a score that is markedly higher than our reported mean of M = 4.2 (SD = 2.8). This notable difference suggests that, while the emotional impact is significant in both groups, patients in our study reported a lower intensity of negative emotional representation compared to the rescaled scores of the earlier study; this discrepancy warrants further investigation. Furthermore, both studies are aligned in showing a significant psychological burden related to a lack of perceived mastery. The low “Treatment Control” and “Personal Control” reported by Cartwright et al. convert to 3.05 and 3.0, respectively, results that support our own low control scores (“Treatment Control” M = 2.9, SD = 2.1 and “Personal Control” M = 6.0, SD = 2.5). Finally, our sample reported significantly higher “Illness Coherence” (M = 7.3, SD = 2.5 vs. converted 4.6 from 2.84 ± 1.2), suggesting a potentially better intellectual understanding and clarity regarding the condition within our sample. This consistency across the control and impact domains, despite the temporal gap, underscores the urgency of developing coping strategies aimed at reinforcing self-efficacy and illness mastery within this patient population

(Cartwright et al., 2009). Our data overall suggest that there are some links between OCD symptoms, IP, and ED among these patients. While we found strong correlations between IP, OCD symptomatology, and ED, the mediation analysis provides a more nuanced understanding of these relationships. Specifically, while the total effect of IP on OCD symptoms was significant, the direct effect was not significant after accounting for ED. This fully mediated relationship suggests that, in the context of AA, a negative IP could amplify feelings of anxiety, frustration, or sadness due to the psychosocial and emotional challenges posed by the condition. These resulting feelings of ED, in turn, can fuel obsessive thoughts and compulsive behaviors as individuals attempt to manage or alleviate their discomfort (Sookman & Pinard, 2002). For instance, someone with a negative view of their illness might experience heightened anxiety, which could then manifest as ritualistic behaviors as a maladaptive coping mechanism (Sharpe et al., 2022). The significant indirect effect we observed indicates that ED acts as exclusive pathway through which IP contributes to OCD symptomatology. For example, a person perceiving their illness negatively might first experience increased ED, which subsequently exacerbates their OCD symptoms. Therefore, it is not the IP directly driving OCD symptoms in our model, but rather its impact on ED that then leads to increased OCD behaviors. This finding of full mediation is key to understanding the psychopathology in this patient group.

### 4.1. Clinical and Research Implications

Based on the identified mechanism, where maladaptive IP significantly contributes to ED, which in turn leads to the exacerbation of OCD symptoms in this patient sample, clinical interventions should target the entire pathway. Specifically, interventions designed for patients with AA exhibiting OCD symptomatology should focus on cognitive restructuring of negative IP and the subsequent effective management of ED. Addressing negative IP might be a way to reduce ED, which in turn could alleviate OCD symptom. There is a growing body of evidence in the literature that highlights the importance and benefit of psychotherapeutic interventions on IP and ED (Dalili & Bayazi, 2019; Sawyer et al., 2019; Striberger et al., 2021). Conversely, well-established intervention protocols for OCD symptoms have been available (Külz et al., 2019; Reid et al., 2021) and it is reasonable to assume that could be beneficially applied to patients with AA who exhibit OCD symptoms. These psychological interventions aim to modify maladaptive beliefs, reduce negative emotional impact, and promote more effective coping strategies (Goulding et al., 2010; Maloh et al., 2023). For example, psychoeducational interventions are helpful in providing accurate and comprehensible information about the illness helping patients in develop a realistic understanding of their condition (Burke et al., 2024; Tomé-Pires et al., 2023). Recently, interventions such as Cognitive Behavioral Therapy, Acceptance and Commitment Therapy and Compassion-Focused Therapy are being also increasingly considered for patients with chronic diseases (McCracken et al., 2022; Zarotti et al., 2023). In this context, these approaches aim to modify maladaptive beliefs and negative thoughts that influence IP and ED. Cognitive restructuring techniques help patients identify and challenge irrational thoughts, adjusting them with more balanced and adaptive ones (Andreae et al., 2021; Ma et al., 2020). These approaches can be beneficial also for patients who have a negative self-perception or who feel guilt or shame due to the characteristics of the illness experienced by this patient group (Hughes et al., 2017). However, it is prudent to emphasize that a substantial body of literature documenting their efficacy in dermatological patients, particularly those with AA, is currently lacking. The effectiveness of these interventions may vary depending on the individual characteristics of the patient, the nature of the illness, and the specific context.

### 4.2. Limitations

Some limitations of the present study should be acknowledged. Firstly, the cross-sectional nature of the study design limits the ability to draw causal inferences. Longitudinal studies are needed. Secondly, the reliance on self-report measures may introduce bias. Moreover, in this study, a crucial methodological note must be established regarding the assessment of psychological comorbidity. Given the limitations of time and setting inherent to dermatological research, our study employs a validated screening instrument for the assessment of OCD symptoms, rather than a structured diagnostic clinical interview. Consequently, while our findings can effectively identify the presence and severity of obsessive–compulsive spectrum symptomatology—including subclinical levels—we refrain from drawing conclusions about the definitive prevalence of the full clinical diagnosis (OCD proper) according to established DSM or ICD criteria.

Thirdly, the sample consisted of outpatients attending a specialist clinic for AA, which may limit the generalizability; moreover, the ability to generalize findings is also constrained by the relatively small, localized nature of the convenience sample. The exclusion of individuals with a history of psychiatric disorders may have resulted in a sample with lower levels of psychopathology compared to the general population of individuals with AA. Future research should provide a more comprehensive understanding of the complex interplay between AA and OCD symptomatology.

## 5. Conclusions

This study highlights the significant interplay between IP, ED, and OCD symptomatology in individuals with AA. The findings stressed the importance of considering these psychological factors in the comprehensive management of AA, as they contribute to the overall burden of the condition and impact patients’ QoL.

## Figures and Tables

**Table 1:** Sociodemographic and clinical features of the study sample *.

Variable	Categories	N * Or Means	% Or SD
Overall		135	100.0
Sex	Male	39	28.9
	Female	96	71.1
Age (years)	m; ±SD	38.7	13.4
BMI	<25	83	68.6
	25–29	26	21.5
	30+	12	9.9
Education	<14	85	64.9
(years)	14+	46	35.1
Marital	In relationship	67	56.3
status	Single	52	43.7
Smoking	Ex-No	73	54.1
	Yes	62	23.8
Alcohol	No/Occasionally	91	67.9
	Yes	43	32.1
Insomnia	No	2	1.5
	Yes	132	98.5
		m	±SD
SALT ^1^		59	49.6
Brief IPQ			
	consequences	7.1	2.4
	timeline	6.5	2.5
	personal control	6.0	2.5
	treatment control	2.9	2.1
	identity	5.6	3.0
	illness coherence	7.3	2.5
	emotional representation	4.2	2.8
	illness concern	7.2	2.6
DASS-21 ^1^	Depression	18	13.7
	Anxiety	7	5.5
	Stress	127	95.5

* Total may vary due to missing values; m = mean; ±SD = standard deviation. SALT = Severity of Alopecia Tool; ^1^ frequencies and percentages of observations categorized from “severe” to “extremely severe” (Bottesi et al., 2015; Wyrwich et al., 2020).

**Table 2:** Association between sociodemographic features and the clinical severity of the examined variables.

	Age	BMI	SALT	DASS-21 Total Score	DASS-21 Anxiety	DASS-21 Depression	DASS-21 Stress	B-IPQ
BMI	0.280 **							
SALT	−0.002	0.082						
DASS-21 total score	−0.102	−0.046	0.164					
DASS-21 anxiety	−0.001	−0.025	0.092	0.541 **				
DASS-21 depression	−0.022	−0.056	0.189 *	0.658 **	0.411 **			
DASS-21 stress	0.001	0.100	0.047	0.301 **	0.054	0.088		
B-IPQ-R	0.062	−0.098	−0.043	0.354 **	0.163	0.180 *	0.065	
OCI-R	−0.122	−0.174	0.085	0.559 **	0.233 **	0.383 **	0.100	0.251 **

** *p* < 0.01; * *p* < 0.05.

## Data Availability

The data that support the findings of this study are available from the corresponding author, [T.S.], upon reasonable request.

## References

[B1-behavsci-16-00092] Afkham Ebrahimi A., Salehi M., Kafian Tafti A. (2007). Obsessive-compulsive disorder in dermatology outpatients. International Journal of Psychiatry in Clinical Practice.

[B2-behavsci-16-00092] Aguilar-Duran S., Ahmed A., Taylor R., Bewley A. (2014). How to set up a psychodermatology clinic. Clinical and Experimental Dermatology.

[B3-behavsci-16-00092] Andreae S. J., Andreae L. J., Richman J. S., Cherrington A. L., Safford M. M. (2021). Peer-delivered cognitive behavioral therapy-based intervention reduced depression and stress in community dwelling adults with diabetes and chronic pain: A cluster randomized trial. Annals of Behavioral Medicine.

[B4-behavsci-16-00092] Baron R. M., Kenny D. A. (1986). The moderator–mediator variable distinction in social psychological research: Conceptual, strategic, and statistical considerations. Journal of Personality and Social Psychology.

[B5-behavsci-16-00092] Bertolini M., McElwee K., Gilhar A., Bulfone-Paus S., Paus R. (2020). Hair follicle immune privilege and its collapse in Alopecia Areata. Experimental Dermatology.

[B6-behavsci-16-00092] Bokor G., Anderson P. D. (2014). Obsessive–compulsive disorder. Journal of Pharmacy Practice.

[B7-behavsci-16-00092] Bottesi G., Ghisi M., Altoè G., Conforti E., Melli G., Sica C. (2015). The Italian version of the depression anxiety stress scales-21: Factor structure and psychometric properties on community and clinical samples. Comprehensive Psychiatry.

[B8-behavsci-16-00092] Burke A., Davoren M. P., Arensman E., Harrington J. M. (2024). Psychoeducational interventions for people living with chronic communicable disease: A systematic review. BMJ Open.

[B9-behavsci-16-00092] Burns L. J., Mesinkovska N., Kranz D., Ellison A., Senna M. M. (2020). Cumulative life course impairment of Alopecia Areata. International Journal of Trichology.

[B10-behavsci-16-00092] Caldarola G., Raimondi G., Samela T., Pinto L., Pampaloni F., Starace M. V. R., Diluvio L., Dall’Oglio F., Vagnozzi E., de Felici del Giudice M. B., Balestri R., Ambrogio F., Girolomoni G., Riva S. F., Moro F., Atzori L., Gallo G., Ribero S., Simonetti O., Peris K. (2024). Assessing a measure for quality of life in patients with severe Alopecia Areata: A multicentric Italian study. Frontiers in Public Health.

[B11-behavsci-16-00092] Caravaca-Sánchez F., Aizpurua E., Wolff N., Ricarte J. J. (2025). Emotional distress in incarcerated populations: Factor structure of the spanish version of the DASS-21. Criminal Justice and Behavior.

[B12-behavsci-16-00092] Cartwright T., Endean N., Porter A. (2009). Illness perceptions, coping and quality of life in patients with alopecia. British Journal of Dermatology.

[B13-behavsci-16-00092] Cervin M. (2023). Obsessive-compulsive disorder: Diagnosis, clinical features, nosology, and epidemiology. Psychiatric Clinics.

[B14-behavsci-16-00092] Chu S. Y., Chen Y. J., Tseng W. C., Lin M. W., Chen T. J., Hwang C. Y., Chen C. C., Lee D. D., Chang Y. T., Wang W. J., Liu H. N. (2012). Psychiatric comorbidities in patients with Alopecia Areata in Taiwan: A case–control study. British Journal of Dermatology.

[B15-behavsci-16-00092] Cohen J. (1977). Statistical power analysis for the behavioral sciences.

[B16-behavsci-16-00092] Dalili Z., Bayazi M. H. (2019). The effectiveness of mindfulness-based cognitive therapy on the illness perception and psychological symptoms in patients with rheumatoid arthritis. Complementary Therapies in Clinical Practice.

[B17-behavsci-16-00092] Demet M. M., Deveci A., Taskin E. O., Turel Ermertcan A., Yurtsever F., Deniz F., Bayraktar D., Ozturkcan S. (2005). Obsessive–compulsive disorder in a dermatology outpatient clinic. General Hospital Psychiatry.

[B18-behavsci-16-00092] Emilsson M., Berndtsson I., Gustafsson P. A., Horne R., Marteinsdottir I. (2020). Reliability and validation of Swedish translation of Beliefs about Medication Specific (BMQ-Specific) and Brief Illness Perception Questionnaire (B-IPQ) for use in adolescents with attention-deficit hyperactivity disorder. Nordic Journal of Psychiatry.

[B19-behavsci-16-00092] Fawcett E., Power H., Fawcett J. (2020). Women are at greater risk of OCD than men: A meta-analytic review of OCD prevalence worldwide. The Journal of clinical psychiatry.

[B20-behavsci-16-00092] Ferreira B., Vulink N., Mostaghimi L., Jafferany M., Balieva F., Gieler U., Poot F., Reich A., Romanov D., Szepietowski J. C., Tomas-Aragones L., Campos R., Tausk F., Zipser M., Bewley A., Misery L. (2024). Correction to ‘classification of psychodermatological disorders: Proposal of a new international classification’. Journal of the European Academy of Dermatology and Venereology.

[B21-behavsci-16-00092] Fineberg N. A., O’Doherty C., Rajagopal S., Reddy K., Banks A., Gale T. M. (2003). How common is obsessive-compulsive disorder in a dermatology outpatient clinic?. Journal of Clinical Psychiatry.

[B22-behavsci-16-00092] Finlay A. Y., Chernyshov P. V., Tomas Aragones L., Bewley A., Svensson A., Manolache L., Marron S., Suru A., Sampogna F., Salek M. S., Poot F. (2021). Methods to improve quality of life, beyond medicines. Position statement of the european academy of dermatology and venereology task force on quality of life and patient oriented outcomes. Journal of the European Academy of Dermatology and Venereology.

[B23-behavsci-16-00092] Firooz A., Firoozabadi M. R., Ghazisaidi B., Dowlati Y. (2005). Concepts of patients with Alopecia Areata about their disease. BMC Dermatology.

[B24-behavsci-16-00092] Foxwell R., Morley C., Frizelle D. (2013). Illness perceptions, mood and quality of life: A systematic review of coronary heart disease patients. Journal of Psychosomatic Research.

[B25-behavsci-16-00092] Goulding L., Furze G., Birks Y. (2010). Randomized controlled trials of interventions to change maladaptive illness beliefs in people with coronary heart disease: Systematic review. Journal of Advanced Nursing.

[B26-behavsci-16-00092] Hallegraeff J. M., van der Schans C. P., Krijnen W. P., de Greef M. H. G. (2013). Measurement of acute nonspecific low back pain perception in primary care physical therapy: Reliability and validity of the brief illness perception questionnaire. BMC Musculoskeletal Disorders.

[B27-behavsci-16-00092] Harrison J. E., Weber S., Jakob R., Chute C. G. (2021). ICD-11: An international classification of diseases for the twenty-first century. BMC Medical Informatics and Decision Making.

[B28-behavsci-16-00092] Hayes A. F. (2013). Introduction to mediation, moderation, and conditional process analysis: A regression-based approach.

[B29-behavsci-16-00092] Hemphill J. F. (2003). Interpreting the magnitudes of correlation coefficients. American Psychologist.

[B30-behavsci-16-00092] Henry J. D., Crawford J. R. (2005). The short-form version of the Depression Anxiety Stress Scales (DASS-21): Construct validity and normative data in a large non-clinical sample. British Journal of Clinical Psychology.

[B31-behavsci-16-00092] Hughes L. S., Clark J., Colclough J. A., Dale E., McMillan D. (2017). Acceptance and commitment therapy (ACT) for chronic pain: A systematic review and meta-analyses. The Clinical Journal of Pain.

[B32-behavsci-16-00092] IBM Corp (2019). IBM SPSS statistics for windows.

[B33-behavsci-16-00092] Jaltuszewska S., Chojnacka-Szawlowska G., Majkowicz M., Zdonczyk S., Homenda W., Hebel K. (2023). Illness perception and the severity of depression and anxiety symptoms in patients with multimorbidity: Observational cohort studies. Journal of Clinical Medicine.

[B34-behavsci-16-00092] Kline P. (1993). The handbook of psychological testing.

[B35-behavsci-16-00092] Külz A. K., Landmann S., Cludius B., Rose N., Heidenreich T., Jelinek L., Alsleben H., Wahl K., Philipsen A., Voderholzer U., Maier J. G., Moritz S. (2019). Mindfulness-based cognitive therapy (MBCT) in patients with obsessive–compulsive disorder (OCD) and residual symptoms after cognitive behavioral therapy (CBT): A randomized controlled trial. European Archives of Psychiatry and Clinical Neuroscience.

[B36-behavsci-16-00092] Lauron S., Plasse C., Vaysset M., Pereira B., D’Incan M., Rondepierre F., Jalenques I. (2023). Prevalence and odds of depressive and anxiety disorders and symptoms in children and adults with Alopecia Areata: A systematic review and meta-analysis. JAMA Dermatology.

[B37-behavsci-16-00092] Lee H. H., Gwillim E., Patel K. R., Hua T., Rastogi S., Ibler E., Silverberg J. I. (2020). Epidemiology of Alopecia Areata, ophiasis, totalis, and universalis: A systematic review and meta-analysis. Journal of the American Academy of Dermatology.

[B38-behavsci-16-00092] Lee S., Lee H., Lee C. H., Lee W. S. (2019). Comorbidities in Alopecia Areata: A systematic review and meta-analysis. Journal of the American Academy of Dermatology.

[B39-behavsci-16-00092] Leventhal H., Benyamini Y., Brownlee S., Diefenbach M., Leventhal E. A., Patrick-Miller L., Robitaille C. (1997). Illness representations: Theoretical foundations. Perceptions of health and illness: Current research and applications..

[B40-behavsci-16-00092] Lintzeri D. A., Constantinou A., Hillmann K., Ghoreschi K., Vogt A., Blume-Peytavi U. (2022). Alopecia Areata—Current understanding and management. Journal der Deutschen Dermatologischen Gesellschaft.

[B41-behavsci-16-00092] Lyakhovitsky A., Aronovich A., Gilboa S., Baum S., Barzilai A. (2019). Alopecia Areata: A long-term follow-up study of 104 patients. Journal of the European Academy of Dermatology and Venereology.

[B42-behavsci-16-00092] Ma R.-C., Yin Y.-Y., Wang Y.-Q., Liu X., Xie J. (2020). Effectiveness of cognitive behavioural therapy for chronic obstructive pulmonary disease patients: A systematic review and meta-analysis. Complementary Therapies in Clinical Practice.

[B43-behavsci-16-00092] Macbeth A. E., Holmes S., Harries M., Chiu W. S., Tziotzios C., de Lusignan S., Messenger A. G., Thompson A. R. (2022). The associated burden of mental health conditions in Alopecia Areata: A population-based study in UK primary care*. British Journal of Dermatology.

[B44-behavsci-16-00092] Maloh J., Engel T., Natarelli N., Nong Y., Zufall A., Sivamani R. K. (2023). Systematic review of psychological interventions for quality of life, mental health, and hair growth in Alopecia Areata and scarring alopecia. Journal of Clinical Medicine.

[B45-behavsci-16-00092] Marchetti I., Chiri L. R., Ghisi M., Sica C. (2010). Obsessive-compulsive inventory-revised (OCI-R): Presentation and instructions for use in Italy. Psicoterapia Cognitiva e Comportamentale.

[B46-behavsci-16-00092] Mavrogiorgou P., Bader A., Stockfleth E., Juckel G. (2015). Obsessive-compulsive disorder in dermatology. JDDG: Journal der Deutschen Dermatologischen Gesellschaft.

[B47-behavsci-16-00092] Maxwell S. E., Cole D. A., Mitchell M. A. (2011). Bias in cross-sectional analyses of longitudinal mediation: Partial and complete mediation under an autoregressive model. Multivariate Behav Res.

[B48-behavsci-16-00092] McCracken L. M., Yu L., Vowles K. E. (2022). New generation psychological treatments in chronic pain. BMJ.

[B49-behavsci-16-00092] Mesinkovska N., Craiglow B., Ball S. G., Morrow P., Smith S. G., Pierce E., Shapiro J. (2023). The invisible impact of a visible disease: Psychosocial impact of Alopecia Areata. Dermatology and Therapy.

[B50-behavsci-16-00092] Mostaghimi A., Napatalung L., Sikirica V., Winnette R., Xenakis J., Zwillich S. H., Gorsh B. (2021). Patient perspectives of the social, emotional and functional impact of Alopecia Areata: A systematic literature review. Dermatology and Therapy.

[B51-behavsci-16-00092] Nasimi M., Abedini R., Ghandi N., Manuchehr F., Kazemzadeh Houjaghan A., Shakoei S. (2021). Illness perception in patients with Alopecia Areata under topical immunotherapy. Dermatologic Therapy.

[B52-behavsci-16-00092] O’Connor S., Hevey D., O’Keeffe F. (2023). Illness perceptions, coping, health-related quality of life and psychological outcomes in cervical dystonia. Journal of Clinical Psychology in Medical Settings.

[B53-behavsci-16-00092] Okhovat J.-P., Marks D. H., Manatis-Lornell A., Hagigeorges D., Locascio J. J., Senna M. M. (2023). Association between Alopecia Areata, anxiety, and depression: A systematic review and meta-analysis. Journal of the American Academy of Dermatology.

[B54-behavsci-16-00092] Olsen E. A., Hordinsky M. K., Price V. H., Roberts J. L., Shapiro J., Canfield D., Duvic M., King L. E., McMichael A. J., Randall V. A., Turner M. L., Sperling L., Whiting D. A., Norris D. (2004). Alopecia Areata investigational assessment guidelines—Part II. National Alopecia Areata foundation. Journal of the American Academy of Dermatology.

[B55-behavsci-16-00092] Osman A., Wong J. L., Bagge C. L., Freedenthal S., Gutierrez P. M., Lozano G. (2012). The depression anxiety stress Scales—21 (DASS-21): Further examination of dimensions, scale reliability, and correlates. Journal of Clinical Psychology.

[B56-behavsci-16-00092] Pain D., Angelino E., Miglioretti M. (2006). Sviluppo della versione italiana del Brief-IPQ (Illness Perception Questionnaire, short version), strumento psicometrico per lo studio delle Rappresentazioni di Malattia.

[B57-behavsci-16-00092] Panebianco A., Sampogna F., Iemboli M. L., Sobrino L., Andreoli E., Antinone V., Mazzanti C., Abeni D. (2018). A screening programme for dermatologists as a guide to request psychological consultation in routine clinical practice. European Journal of Dermatology.

[B58-behavsci-16-00092] Pelzer C., Iorizzo M. (2024). Alopecia Areata of the nails: Diagnosis and management. Journal of Clinical Medicine.

[B59-behavsci-16-00092] Petukhova L., Duvic M., Hordinsky M., Norris D., Price V., Shimomura Y., Kim H., Singh P., Lee A., Chen W. V., Meyer K. C., Paus R., Jahoda C. A., Amos C. I., Gregersen P. K., Christiano A. M. (2010). Genome-wide association study in Alopecia Areata implicates both innate and adaptive immunity. Nature.

[B60-behavsci-16-00092] Picardi A., Pasquini P., Abeni D., Fassone G., Mazzotti E., Fava G. A. (2005). Psychosomatic assessment of skin diseases in clinical practice. Psychotherapy and Psychosomatics.

[B61-behavsci-16-00092] Preacher K. J., Hayes A. F. (2008). Asymptotic and resampling strategies for assessing and comparing indirect effects in multiple mediator models. Behavior Research Methods.

[B62-behavsci-16-00092] Reid J. E., Laws K. R., Drummond L., Vismara M., Grancini B., Mpavaenda D., Fineberg N. A. (2021). Cognitive behavioural therapy with exposure and response prevention in the treatment of obsessive-compulsive disorder: A systematic review and meta-analysis of randomised controlled trials. Comprehensive Psychiatry.

[B63-behavsci-16-00092] Rencz F., Gulácsi L., Péntek M., Wikonkál N., Baji P., Brodszky V. (2016). Alopecia Areata and health-related quality of life: A systematic review and meta-analysis. British Journal of Dermatology.

[B64-behavsci-16-00092] Roest Y. B. M., van Middendorp H. T., Evers A. W. M., van de Kerkhof P. C. M., Pasch M. C. (2018). Nail involvement in Alopecia Areata: A questionnaire-based survey on clinical signs, impact on quality of life and review of the literature. Acta Dermato-Venereologica.

[B65-behavsci-16-00092] Rudnicka L., Arenbergerova M., Grimalt R., Ioannides D., Katoulis A. C., Lazaridou E., Olszewska M., Ovcharenko Y. S., Piraccini B. M., Prohic A., Rakowska A., Reygagne P., Richard M. A., Soares R. O., Starace M., Vañó-Galvan S., Waskiel-Burnat A. (2024). European expert consensus statement on the systemic treatment of Alopecia Areata. Journal of the European Academy of Dermatology and Venereology.

[B66-behavsci-16-00092] Samela T., Cordella G., Antinone V., Sarandrea P., Giampetruzzi A. R., Abeni D. (2022). The use of SCL-k-9 to measure general psychopathology in women and men with skin conditions. Frontiers in Psychology.

[B67-behavsci-16-00092] Samela T., Innamorati M., Lester D., Raimondi G., Giupponi G., Imperatori C., Contardi A., Fabbricatore M. (2021). The association between adult ADHD and food addiction: A mediation analysis. Appetite.

[B68-behavsci-16-00092] Sawyer A. T., Harris S. L., Koenig H. G. (2019). Illness perception and high readmission health outcomes. Health Psychology Open.

[B69-behavsci-16-00092] Sharpe L., Todd J., Scott A., Gatzounis R., Menzies R. E., Meulders A. (2022). Safety behaviours or safety precautions? The role of subtle avoidance in anxiety disorders in the context of chronic physical illness. Clinical Psychology Review.

[B70-behavsci-16-00092] Sheikhmoonesi F., Hajheidari Z., Masoudzadeh A., Mohammadpour R. A., Mozaffari M. (2014). Prevalence and severity of obsessive-compulsive disorder and their relationships with dermatological diseases. Acta Medica Iranica.

[B71-behavsci-16-00092] Solmaz N., Ilhan N., Bulut H. M. (2021). The effect of illness perception on life quality in psoriasis patients. Psychology, Health & Medicine.

[B72-behavsci-16-00092] Sookman D., Pinard G., Frost R. O., Steketee G. (2002). Chapter 5—Overestimation of threat and intolerance of uncertainty in obsessive compulsive disorder. Cognitive approaches to obsessions and compulsions.

[B73-behavsci-16-00092] Streiner D. L. (2003). Starting at the beginning: An introduction to coefficient alpha and internal consistency. Journal of Personality Assessment.

[B74-behavsci-16-00092] Striberger R., Axelsson M., Zarrouk M., Kumlien C. (2021). Illness perceptions in patients with peripheral arterial disease: A systematic review of qualitative studies. International Journal of Nursing Studies.

[B75-behavsci-16-00092] Talaei A., Nahidi Y., Kardan G., Jarahi L., Aminzadeh B., Jahed Taherani H., Nahidi M., Ziaee M. (2017). Temperament-character profile and psychopathologies in patients with Alopecia Areata. The Journal of General Psychology.

[B76-behavsci-16-00092] Titeca G., Goudetsidis L., Francq B., Sampogna F., Gieler U., Tomas-Aragones L., Lien L., Jemec G. B. E., Misery L., Szabo C., Linder D., Evers A. W. M., Halvorsen J. A., Balieva F., Szepietowski J., Romanov D., Marron S. E., Altunay I. K., Finlay A. Y., Poot F. (2020). ‘The psychosocial burden of Alopecia Areata and androgenetica’: A cross-sectional multicentre study among dermatological out-patients in 13 European countries. Journal of the European Academy of Dermatology and Venereology.

[B77-behavsci-16-00092] Tomé-Pires C., Aragonès E., Rambla C., López-Cortacans G., Sánchez-Rodríguez E., Caballero A., Miró J. (2023). Perceived barriers, facilitators and usefulness of a psychoeducational intervention for individuals with chronic musculoskeletal pain and depression in primary care. Frontiers in Psychology.

[B78-behavsci-16-00092] Toussi A., Barton V. R., Le S. T., Agbai O. N., Kiuru M. (2021). Psychosocial and psychiatric comorbidities and health-related quality of life in Alopecia Areata: A systematic review. Journal of the American Academy of Dermatology.

[B79-behavsci-16-00092] Van Beugen S., Schut C., Kupfer J., Bewley A. P., Finlay A. Y., Gieler U., Thompson A. R., Gracia-Cazaña T., Balieva F., Ferreira B. R., Jemec G. B., Lien L., Misery L., Marron S. E., Ständer S., Zeidler C., Szabó C., Szepietowski J. C., Reich A., Dalgard F. J. (2023). Perceived stigmatization among dermatological outpatients compared with controls: An observational multicentre study in 17 european countries. Acta Dermato-Venereologica.

[B80-behavsci-16-00092] Villasante Fricke A. C., Miteva M. (2015). Epidemiology and burden of Alopecia Areata: A systematic review. Clinical, Cosmetic and Investigational Dermatology.

[B81-behavsci-16-00092] Weinman J., Petrie K. J., Moss-Morris R., Horne R. (1996). The illness perception questionnaire: A new method for assessing the cognitive representation of illness. Psychology & Health.

[B82-behavsci-16-00092] Wyrwich K. W., Kitchen H., Knight S., Aldhouse N. V. J., Macey J., Nunes F. P., Dutronc Y., Mesinkovska N., Ko J. M., King B. A. (2020). The Alopecia Areata investigator global assessment scale: A measure for evaluating clinically meaningful success in clinical trials. British Journal of Dermatology.

[B83-behavsci-16-00092] You H. R., Kim S. J. (2017). Factors associated with severity of Alopecia Areata. Annals of Dermatology.

[B84-behavsci-16-00092] Zarotti N., Eccles F., Broyd A., Longinotti C., Mobley A., Simpson J. (2023). Third wave cognitive behavioural therapies for people with multiple sclerosis: A scoping review. Disability and Rehabilitation.

[B85-behavsci-16-00092] Zhou C., Li X., Wang C., Zhang J. (2021). Alopecia Areata: An update on etiopathogenesis, diagnosis, and management. Clinical Reviews in Allergy & Immunology.

